# A single nucleotide polymorphism in dopamine beta hydroxylase (rs6271(C>T)) is over-represented in inflammatory bowel disease patients and reduces circulating enzyme

**DOI:** 10.1371/journal.pone.0210175

**Published:** 2019-02-28

**Authors:** Eugene Gonzalez-Lopez, Yuka Kawasawa-Imamura, Lijun Zhang, Xuemei Huang, Walter A. Koltun, Matthew D. Coates, Kent E. Vrana

**Affiliations:** 1 Department of Pharmacology, Penn State College of Medicine, Hershey, Pennsylvania, United States of America; 2 Genome Sciences Core Facility, Institute for Personalized Medicine, Pennsylvania State University College of Medicine, Hershey, Pennsylvania; 3 Department of Biochemistry and Molecular Biology, Pennsylvania State University College of Medicine, Hershey, Pennsylvania; 4 Department of Neurology, Pennsylvania State University College of Medicine, Milton S. Hershey Medical Center, Hershey, Pennsylvania, United States of America; 5 Departments of Neurology, Neurosurgery and Radiology, Milton S. Hershey Medical Center, and Kinesiology, Pennsylvania State University, Hershey, Pennsylvania, United States of America; 6 Department of Surgery, Division of Colon and Rectal Surgery, Pennsylvania State University, Hershey, Pennsylvania, United States of America; 7 Department of Medicine, Division of Gastroenterology & Hepatology, Pennsylvania State University Hershey Medical Center, Hershey, Pennsylvania, United States of America; Max-Planck-Institut fur Psychiatrie, GERMANY

## Abstract

Inflammatory bowel diseases (IBD) are associated with altered neuronal regulation of the gastrointestinal (GI) tract and release of norepinephrine (NE). As sympathetic innervation of the GI tract modulates motility, blood flow, and immune function, changes in NE signaling may alter the risk of developing IBD. Dopamine beta-hydroxylase (DβH), the enzyme responsible for NE production, has been suggested to play a critical role in IBD, however the exact mechanism is unknown. We hypothesized that genetic variants of *DβH* could increase the risk of IBD. We performed genetic analysis on 45 IBD patients and 74 controls. IBD patients were screened by targeted exome sequencing and compared with NeuroX *DβH* single nucleotide polymorphism (SNP) genotyping data of the controls. Serum DβH protein levels for 15 IBD patients and 13 controls were evaluated using immunoblots and competitive ELISA. Seven SNPs were observed from DβH targeted exome sequencing in the 45 IBD patients. A single non-synonymous SNP, rs6271 (Arg549Cys), had a significant association with IBD patients; the odds ratio was a 5.6 times higher SNP frequency in IBD patients compared to controls (*p* = 0.002). We also examined the function and availability of the protein in both the IBD and control patients’ sera bearing DβH Arg549Cys. Both control and IBD subjects bearing the heterozygote allele had statistically lower DβH protein levels while the intrinsic enzyme activity was higher. This is the first report of a noradrenergic genetic polymorphism (rs6271; Arg549Cys) associated with IBD. This polymorphism is associated with significantly lower levels of circulating DβH.

## Introduction

The inflammatory bowel diseases (IBD) Crohn’s disease (CD) and ulcerative colitis (UC) are chronic disorders of the gastrointestinal (GI) tract that affect millions of people in the U.S. alone. They are often debilitating illnesses without medical cures that can be very challenging to manage, in part because of our incomplete understanding of the pathophysiology underlying these conditions. A variety of factors that directly affect host immune function and inflammatory control have been implicated in IBD, including alterations in the gut microbiome, other environmental exposures, and genetic influences [1, 2]. As a result, the majority of therapies currently available to treat IBD rely on strategies that directly address inflammatory mediators implicated in the development and perpetuation of IBD [3]. Although these medications can be helpful, many patients are intolerant to them or do not demonstrate a lasting response to even the most potent treatments [4]. Therefore, there is an ongoing need to develop novel strategies to manage IBD.

One promising approach for potential IBD therapy involves neuro-immune modulation of the gut. Alterations in gut-associated nerves and neuronal signaling in IBD have been described for several decades. Intestinal nerve fiber density may be altered in both CD and UC [5, 6]. Many key neuroendocrine signaling factors appear to change in IBD, including norepinephrine (NE) and other factors associated with sympathetic nervous signaling within the gut [7–11]. Every major division of the extrinsic and intrinsic nervous system innervating the gut has demonstrated alterations in one or more of the factors above in animal models of intestinal inflammation and/or in human IBD tissue samples [12, 13]. The sympathetic nervous system has drawn recent attention for its potential role in IBD given its intimate interaction with gut-associated immune cells and structures. Sympatho-noradrenergic nerve fibers innervate multiple layers of the gut, including the myenteric and submucosal plexuses along with the serosa and mucosa, and their neurites can be found immediately juxtaposed to key immune tissues and cells within the submucosal and mucosal layers (including antigen presenting cells, lymphocytes, and plasma cells) that are critical for the acute and chronic phases of IBD [13]. Noradrenergic receptors (of all subtypes) can be found on all of the major immune cell types within the gut [14–16]. It is therefore not surprising that NE can exert a profound impact on intestinal inflammatory activity, acting as either a pro- or anti-inflammatory influence in IBD [16, 17]. Evidence from animal studies shows that a reduction in noradrenergic signaling within the gut, through either chemical or surgical sympathectomy, can result in exacerbation of later phases of intestinal inflammation [15]. Exactly how alterations in NE levels within the gut affect these changes and why they occur in IBD is still unclear. However, changes to dopamine beta-hydroxylase (DβH), the rate-limiting enzyme required for the production of NE from dopamine, may be critical in this regard.

Recent studies have suggested that the expression of DβH may be increased in colonic tissue of patients with IBD as well as in dextran sodium sulfate (DSS) and trinitrobenzene sulfonic acid (TNBS) models of colitis in rodents [18]. Of note, a large number of single nucleotide polymorphisms (SNPs) have been identified in the gene for DβH and some of these variants have been implicated in altered enzyme localization and activities [19–21]. They have also previously been associated with specific illnesses, including cardiovascular [22–24] and neuropsychiatric disorders [23, 25]. However, little is known about their potential influence in the GI tract. This study is the first to examine the entire coding region of *DβH* to identify SNPs that are linked to IBD. We hypothesized that genetic variants in the *DβH* gene result in altered NE production and/or function that contribute to the pathogenesis of IBD.

## Results

### Study participant demographics

A total of 45 IBD (38.2±3.8 years old) patient samples were obtained from Penn State Hershey Medical Center’s IBD BioBank for Central Pennsylvania along with 74 control subjects (65.1±8.7 years old). None of the controls have ever had IBD in their lifetime. Of the 45 IBD patients, twenty-one (21) participants (10 females (47.6%)) were diagnosed with Crohn’s disease (CD) while 24 (12 females (50.0%)) were diagnosed with ulcerative colitis (UC). While the control group had a higher mean age, they were derived from the same geographic population (central Pennsylvania) as the IBD cohort and had no history of IBD.

### Exonic coverage of DβH from targeted exome sequencing

**Fig 1:**
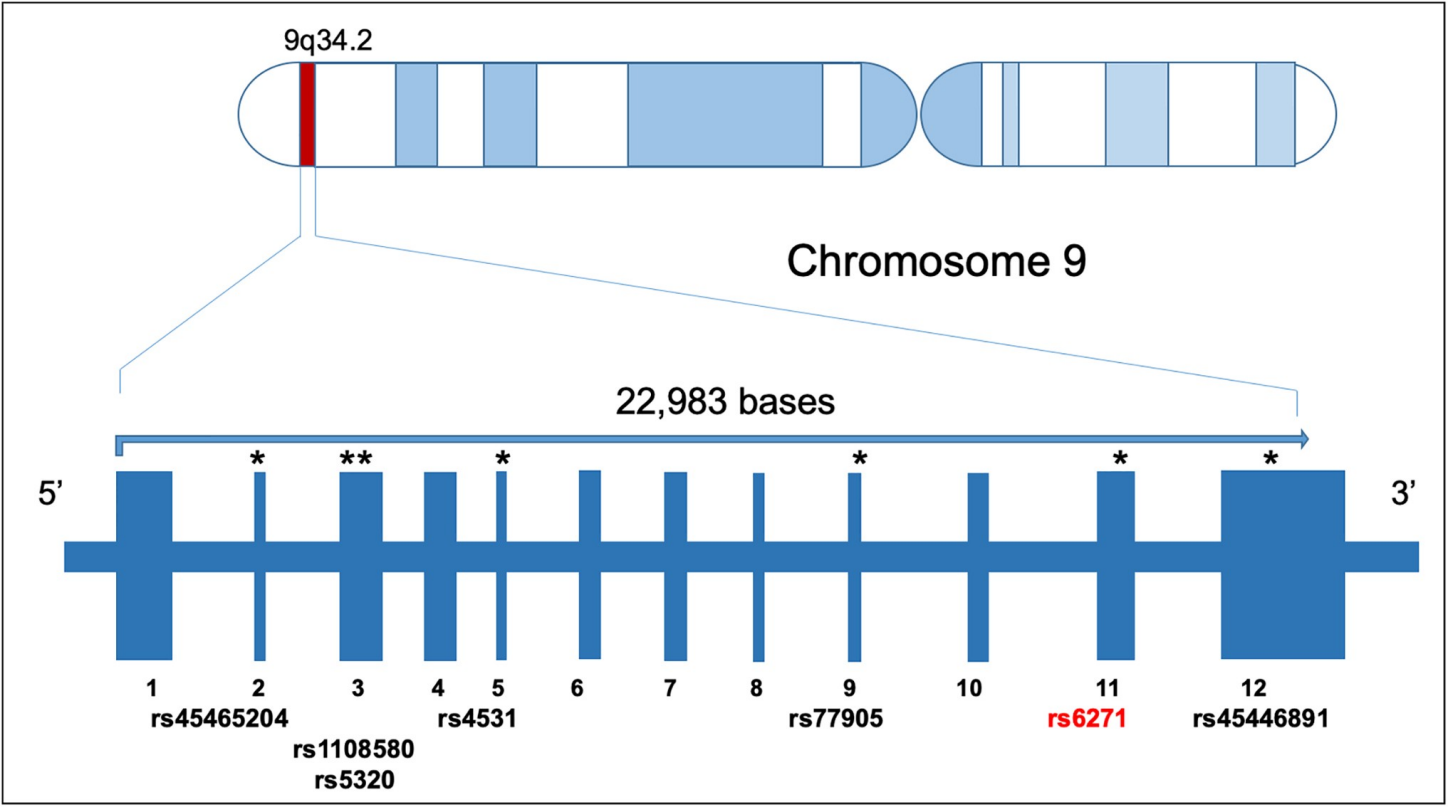
Map of DβH gene with all exonic SNPs and rs# observed in the targeted deep sequencing of IBD patients.

Overall, the mean read depth for the targeted DβH exome sequence was 85x, with 79% of the exome covered at least 59x. A total of seven exonic single nucleotide polymorphisms (SNPs) were obtained in the DΒH gene from the targeted deep sequencing of the 45 IBD patients by the GATK and SAM-tools pipeline (Fig 1). An independent orthogonal assessment of SNP TaqMan genotyping was also used to confirm the deep sequencing data. DβH genotypes were analyzed by TaqMan and matched 100% to the targeted exome sequencing results.

### SNP association analysis: Comparing IBD patients with control

Table 1 presents the odds ratio of all the SNPs observed in the deep sequenced 45 IBD patients compared to 74 controls (ranked from the most statistically-significant to least). One SNP (rs6271) had a significant association in IBD patients when compared to control (p<0.0001; Fisher’s exact test). Allelic frequency (AF) distribution of the Arg549Cys polymorphism was significantly different between 45 IBD and controls when analyzed using a chi-squared test (p<0.0001). The overall AF in the controls was similar to published values (0.02) (https://www.ncbi.nlm.nih.gov/snp/rs6271). The AF for the rs6271 in the IBD group was 5.6 times that of the controls (0.27).

**Table 1:** Single nucleotide polymorphism (SNP) association analysis comparing IBD patients with control.

RS#	Residue change	Minor allele	Risk Allele	MAF-IBD N = 45	MAF-Control N = 74	Odds Ratio (OR)	95% Confidence Interval (Cl)	p-Value
rs6271*	R549C	C	T	13.3	2.7	5.5385	1.7281 to 17.7501	0.0040
**rs45446891**	N578N	C	T	1.11	0	4.9777	0.2006 to 123.5139	0.3273
**rs1108580**	E162E	A	G	47.8	51.3	0.8667	0.5130 to 1.4643	0.5933
rs77905*	T470T	G	A	48.9	45.7	1.1253	0.6659 to 1.9018	0.6592
**rs3025380**	G88A	G	C	0	0.67	0.5433	0.0219 to 13.4801	0.7096
**rs77273740**	R79W	C	T	0	0.67	0.5433	0.0219 to 13.4801	0.7096
**Rs5324**	D290N	G	A	0	0.67	0.5433	0.0219 to 13.4801	0.7096
**rs5320**	A211T	G	A	3.33	4.05	0.8161	0.1990 to 3.3473	0.7778
**rs4531**	A318S	G	T	5.56	4.73	1.1849	0.3645 to 3.8514	0.7779
**rs45465204**	N201S	A	G	1.1	1.35	0.8202	0.0733 to 9.1775	0.8722

IBD patient Identified by targeted exome sequencing

Control patient Identified by NeuroX SNP array analysis

* Confirmed by TaqMan genotyping assay

As further confirmation of the allelic enrichment of rs6271 (Arg549Cys), TaqMan genotyping was performed on the deep-sequenced IBD patients, and heathy controls. Table 2 provides a composite analysis of those 45 IBD patient samples along with 74 control samples. The results from the deep-sequencing and TaqMan match 100%. This results in an AF of 13% in IBD compared with 2.6% in controls (and 2.1% in the NCBI database [26]). The resulting odds ratio indicates a 5-fold increase in rs6271 in IBD compared to control.

**Table 2:** Enrichment between rs6271 genotype and clinical cases of IBD.

**Genotype Status**	**IBD (n = 45)**	**Control (n = 74)**	**P-Value for IBD**	**OR for IBD (95% CI)**
Rs6271	N (%)	N (%)		
C/C (Wild-Type) (549^Arg/Arg^)	34 (75%)	70 (95%)	0.0052	0.1771 (.0471-5243)
C/T (Heterozygote) (549^Arg/Cys^)	10 (22%)	4 (5%)	0.0078	5.286 (1.6790–19.09)
T/T (Homozygote SNP) (549^Cys/Cys^)	1 (2%)	0 (0%)	N/A*	
**Allele Frequency Status**	**IBD (n = 90)**	**Control (n = 148)**	**P-Value for IBD**	**OR for IBD (95% CI)**
Rs6271	N (%)	N (%)		
C (Arginine)	78 (87%)	144 (97%)	0.0029	0.1645 (0.052–0.523)
T (Cysteine)	12 (13%)	4 (2.7%)	0.0029	5.5385 (1.917–19.27)

CI = Confidence interval, OR = Odds ratio

*No Statistical Analysis Possible (n = 1)

### Genetic variant rs6271 protein analysis within IBD and control groups

**Fig 2:**
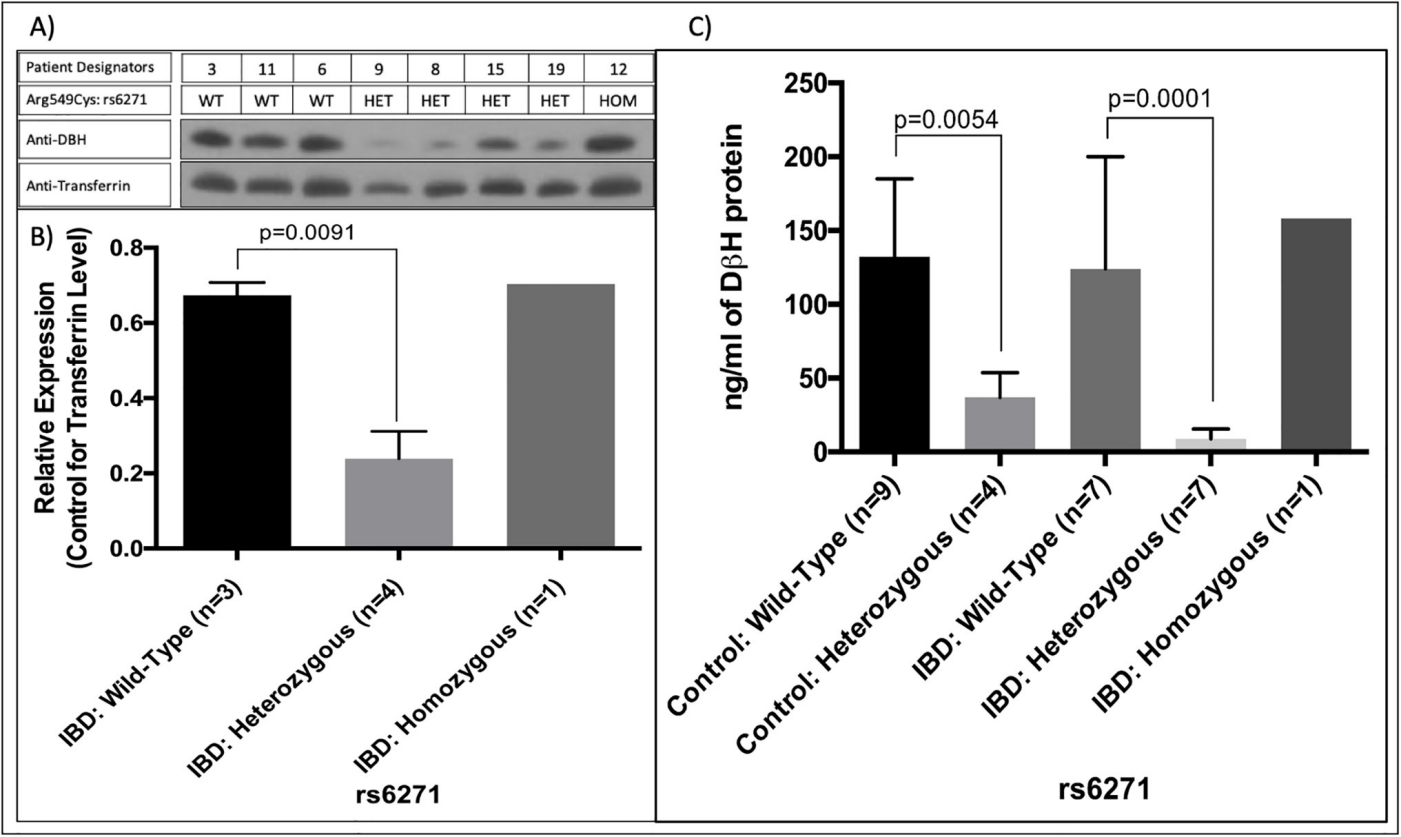
Expression results for DβH in sera from IBD subjects with different genotypes of Arg549Cys. (A) The relative expression levels of DβH among wild-type, heterozygous and homozygous IBD sera. Numbers at the top refer to patient designators. (B) The relative expression levels of DβH controlled for transferrin among wild-type, heterozygous and homozygous IBD serums. Overall comparison of wild-type and heterozygous subject sera are shown as mean±SEM (p = 0.0091; as measured with unpaired t-test). C) The distribution of DβH quantity in both study cohorts as estimated from ELISA assays. Based on the ELISA, heterozygotes for Arg549Cys display lower circulating DβH compared to wildtype in both Control (p = 0.0056) and IBD (p = 0.0001).

Western blot results for the presence of DβH protein in the sera of selected IBD subjects with different genotypes of Arg549Cys (3 wildtype, 4 heterozygous, and 1 homozygous) are presented in Fig 2A. Overall, there is less serum DβH in the heterozygotes (rs6271) than wild-type homozygotes. It should be noted that, given the low allelic frequency for rs6271 (2.7% in controls and 13% in our IBD population), there was only a single homozygote in the IBD population and that the levels of serum DβH protein in this individual were not different than wild-type. Statistical analysis could not be performed on this single data point. Fig 2B shows the expression of DβH in serum with transferrin levels as a loading control.

For rigor and reproducibility, protein quantification of DβH was also performed using competitive ELISA for 15 IBD samples from different genotypes of Arg549Cys (7 wildtype, 7 heterozygous, and 1 homozygous) and 13 controls (9 wildtype, 4 heterozygous). The distribution of DβH quantity in the study cohort as estimated from the standard curve is presented in Fig 2C. Mean serum DβH concentrations in the IBD cohort ranged from 11.5±5.9 ng/ml (heterozygotes; n = 7) to 124.1±76 ng/ml (wild-type homozygotes; n = 7) to 158.3ng/ml for the single homozygous SNP. This analysis in control samples produced the same relationship with lower circulating DβH levels in heterozygotes for Arg549Cys; the control group mean serum DβH concentrations ranged from 34.4±16.7ng/ml (heterozygotes; n = 4) and 129.3±16.5ng/ml (homozygotes wild-type; n = 9).

### Genetic variant rs6271 enzyme activity within IBD and control groups

**Fig 3:**
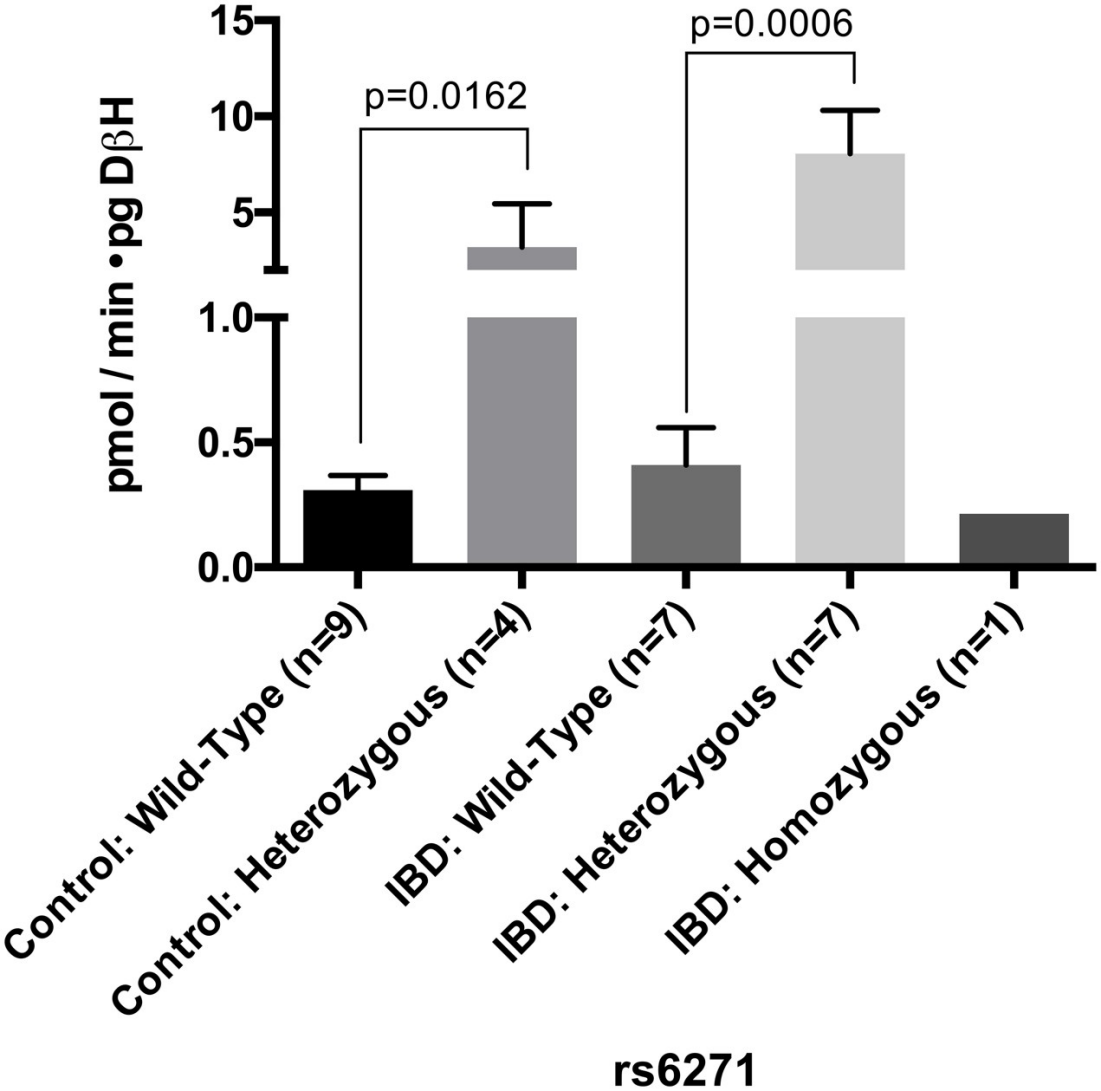
The levels of conversion of tyramine to octopamine in serum samples from IBD and control subjects with different genotypes. Relative activities after normalization to the DΒH expression levels measured by ELISA and represented in Fig 2. Overall comparison of wildtype and heterozygotes subject serum conversion of tyramine to octopamine (normalized to the amount of DΒH present; quantified as pmol/min/pg DβH, measured with an unpaired t-test).

An activity assay, in which tyramine is converted to octopamine, was utilized to measure DβH activity by spectrophotometric measurement of the formation of a co-substrate DMPD cationic radical [27, 28]. Fig 3 shows the amount of tyramine converted to octopamine by equal volumes of a serum sample from wild-type, heterozygous and homozygous subjects. The activity was normalized by the DβH protein concentration (ng/ml) in serum determined by ELISA assay. There was a significant difference between the overall activity within different genotypes in both the IBD group and controls (Fig 3). In both cohorts, the heterozygous subjects had higher utilization of tyramine. Mean serum DβH activity per picogram of DβH in the IBD cohort ranged from 8.1±5.9 pmol/min•pg DβH for heterozygotes to 0.41±0.15 pmol/min•pg DβH for wild-type homozygotes with a *p* = 0.0006. In the control cohort serum, DβH activity was 3.20±4.5 pmol/min•pg DβH for heterozygotes and 0.31±016 pmol/min•pg DβH in wild-type homozygotes with a *p* = 0.0162.

## Discussion

### The rs6271 SNP is associated with increased IBD risk

We have shown a clear enrichment between the presence of the C-to-T single nucleotide polymorphism (SNP) in *DβH* at position 1619 (rs6271) (resulting in a Arg549Cys substitution) and an increased risk of IBD in a Caucasian population. This variant comprised 13% of alleles from IBD patients and only 2.6% of control alleles (OR = 5.6). Interestingly, the allelic frequency in our control population (2.6%) is similar to the global frequency reported by NCBI (2.1%) (https://www.ncbi.nlm.nih.gov/snp/rs6271). We believe this has one or more functionally relevant effects, as our data demonstrate that there is substantially less DβH protein in the serum of IBD patients bearing the heterozygous allele for Arg549Cys in DβH.

### rs6271 SNP and DβH structure

It is possible that the rs6271 genetic variant significantly impacts the structure of the DβH enzyme. Human DβH is comprised of four domains: one dopamine beta-monooxygenase N-terminal (DOMON) domain, two copper catalytic cores and the tetramerization domain. The entire protein is composed of 617 amino acids that include fourteen cysteine residues. Twelve of these residues form six intramolecular disulfide bridges, and the other 2 residues form intermolecular bonds [29]. The DβH tetramer is the active form, whereas no activity is reported in the dimeric or monomeric forms [29]. Computer modeling of DβH by Kapoor et al. (2011) suggests that the Arg549 amino acid residue is exposed on the surface, and its substitution with cysteine may produce non-native disulfide bond formation [30]. This position of Arg549 was subsequently confirmed by Hans and colleagues who described the crystal structure of DβH (deposited in the Protein Data Bank (PDB) under accession code 4ZEL) [29]. Based upon these findings, and our ELISA/western blot data, we believe that the Arg549Cys mutation results in a protein that gets trapped in the cell, either in the ER or in larger aggregates with other proteins in the cell, and is subsequently degraded. Overall, there is dramatically less serum DβH in the heterozygotes (rs6271) than wild-type homozygotes and the single homozygote rs6271 patient identified in this study. However, in both cohorts, the heterozygous subjects had higher inherent specific activity. Interestingly, we believe that there is more to the story, the lower circulating protein suggests altered posttranslational modification, lower stability of the protein and/or lower trafficking. This might affect the stress pathways in the cell and damage the noradrenergic neuron, thereby contributing to the development of IBD by the decrease in NE synthesis, which reduces a potential protective agent in the gastrointestinal tract and elsewhere. It is possible that the higher specific activity of rs6271 heterozygotes may result in an overall decrease in enzyme expression. The observation of increased specific activity in sera therefore warrants further investigation employing cell-based models. Measurements of NE and dopamine in sera/tissue are needed to investigate more fully the role of NE and DβH SNPs in IBD. This will require prospective sample collection as well as genetic considerations involving polymorphisms in metabolic enzymes such as catechol-O-methyltransferase (COMT) and monoamine oxidase (MAO).

### DβH as a potential diagnostic tool

This study describes the first association between a polymorphism in a noradrenergic gene and IBD and offers new insights regarding the influence of genetic factors on the pathogenesis of these disorders. As such, it offers new potential pathways to explore for the improvement of diagnosis and treatment of IBD. This system is also relatively unique in that, among the enzymes that metabolize catecholamines, DβH is localized to large dense core vesicles, where it exists in both soluble and membrane-bound forms [31]. During neurotransmission and adrenal medullary signaling, the soluble form of DβH is released together with NE by exocytosis. As a result, both NE and DβH are readily detectable in serum/plasma and cerebrospinal fluid making them attractive as potential diagnostic tools in this setting [32–34].

### Evaluation and future outlook

These findings support a potential role for sympathetic and noradrenergic signaling in the pathogenesis of IBD. A potential limitation to this study was the relatively small size of our IBD cohort. Although rs6271 is relatively rare, it is possible that we could be witnessing a founder effect in the study group. However, this is less likely because there was a much larger healthy control population derived from the same region that was included in this analysis, which demonstrated an allelic frequency for this SNP that was comparable to that previously reported in the general population. The age of the control population was statistically higher with a mean age of 65.1±8.7. These patients had no history of IBD and, based on their age, are unlikely to develop the disease. Several studies suggest a correlation between age under 40 and incidence and severity of IBD [35, 36]. Also, lower levels of DβH were measured in the serum samples from both controls and IBD patients with the rs6271 polymorphism. We are confident in this finding due to the fact that we utilized two separate methods to quantify protein concentration. While our data contradict what was described in previous studies evaluating intestinal DβH [18], there may be differential expression of this protein in the blood and gut. We did not evaluate intestinal tissue in this study. The association between IBD and rs6271 SNP found in this study does not establish a cause-and-effect relationship between the SNP and these disorders; it is still not clear what functional impact this mutation has inside the cell. Further studies, including evaluation of DβH in intestinal biopsies and modeling these specific changes in cellular/animal studies, are needed to elucidate the exact role of the changes described in our study.

## Methods

### Study participant selection

We obtained clinical and patient survey data from 45 individuals who had consented to take part in a prospective IBD natural history registry and tissue biorepository associated with the IBD Center at the Penn State Hershey Medical Center (approved by the Penn State College of Medicine Institutional Review Board (PRAMSHY98-057)). Materials and information were collected from patients who had undergone a colonoscopy between October 1, 2015, and January 31, 2017. Seventy-four (74) control patients were selected from a large cohort, free of any diagnosed major neurological illness and IBD, currently followed at the Hershey Medical Center Neurology Outpatient Clinic for an ongoing biomarker longitudinal study. Written informed consent was obtained from all control subjects and the study was approved by the Penn State College of Medicine Institutional Review Board (IRB# 40726 for the 74 control patients) and conducted in accordance with the principles of the Declaration of Helsinki.

### Genetic analyses

Blood samples were obtained from 45 IBD patients and 74 controls for DNA analysis.

High-quality genomic DNA was isolated from whole blood using silica-based spin columns QIAmp DNeasy Blood & Tissue Kit (Qiagen, Hilden, Germany). Spectrophotometry was used to quantify DNA and the quality of the isolated material was assessed with an Agilent Bioanalyzer.

#### Next generation deep sequencing

A custom capture oligonucleotide set was designed using the SureDesign platform from Agilent Technologies (SureSelectXT Custom Capture Oligo) against *DβH*. Primers for targeted regions of the promoter (10 kb upstream region from the transcription start site) and 12 exons including 5′ and 3′ untranslated regions (UTRs) of the DβH gene locus (9q34) were used to construct a sequencing library using the KAPA LTP Library Preparation Kit (Kapa Biosystems, Inc., Wilmington, MA) combined with a SureSelectXT Reagent Kit (Agilent Technologies), and sequenced on an Illumina HiSeq 2500 sequencer (Illumina, Inc., San Diego, CA) at read length of paired-end 2x100 bp. Generated reads were aligned to the GRCh37 human reference genome using the Burrows-Wheeler alignment [37]. Variant detection and analysis were performed using the GATK Best Practice for germline SNP/indel finding workflow (Broad Institute). ANNOVAR software [38] was used to annotate the variants and identify synonymous, non-synonymous and deleterious variants for further analysis. For rigor and reproducibility, a TaqMan SNP Genotyping assays designed against the rs6271 and rs77905 SNPs (Life Technologies, Thermo Fisher Scientific, Grand Island, NY) were used to verify the DβH genotype of each patient and control.

#### NeuroX genotyping data acquisition and analysis of control samples

Single-nucleotide polymorphism (SNP) genotyping on 74 control patients population was performed on whole blood DNA samples extracted by the National Institute of Neurological Disorders and Stroke (NINDS) Parkinson’s Disease Biomarkers Program (PDBP) using the Illumina NeuroX array. A total of 269,476 variants were genotyped, and the Genotyping Analysis Module within Genome Studio version 1.9.4 was used to call participant genotypes. We specifically extracted the *DβH* SNP data from the NINDS NeuroX genotyping data.

### DβH protein quantification

#### Immunoblot quantification of DβH in 15 IBD patients and 11 controls

The overall protein concentration in sera was measured using the Bradford Protein Assay (Bio-Rad, Hercules, CA, USA), as described previously [39]. Equal amounts of total protein (20μg) were resolved on a 10 well SDS-PAGE gel. Precast gels (NuPAGE Novex 4–12% Bis-Tris Protein Gels) were obtained from Invitrogen/ThermoFisher Scientific (Grand Island, NY). Western blotting was performed as described previously [39]. A 1:1000 dilution of polyclonal rabbit DβH primary antibody was prepared in 5% BSA in phosphate buffered saline-tween 20 (PBS-TT) to probe the membrane at 4°C overnight (polyclonal DβH antibody was obtained from Cell Signaling Technology, Danvers, MA). The blots were then developed with 1:5000 dilution of an anti-rabbit secondary antibody prepared in 5% dry milk in PBS-TT (HRP-conjugated rabbit secondary antibody was obtained from Cell Signaling Technology, Danvers, MA). An enhanced chemiluminescent (ECL) reagent was employed to visualize the immunoblot signal (Pierce, ThermoFisher Scientific, Grand Island, NY). Transferrin was used as a loading control (monoclonal transferrin primary antibody was obtained from Abcam, Cambridge, MA). ImageJ software [40] was employed to quantify the intensity of each band. The DβH band intensities were normalized to transferrin and the expression levels of DβH in different subjects were compared.

#### Enzyme-Linked Immunosorbent Assay (ELISA) quantification of DβH

DβH levels in the sera samples were also quantified using a Human DβH ELISA Kit (cat no.: CSB-E09653h; Cusabio, Wuhan, P.R. China) with appropriate controls according to the manufacturer’s protocol. The absorbance at 450 nm was measured on a FlexStation 3 plate reader (Molecular Devices), and was plotted against the concentration of standards and the curve was fit by log transformation to a linear fit model. The unknowns were interpolated using GraphPad Prism.

### DβH enzyme activity

#### Enzyme assay by colorimetric method

DΒH activity was measured by a continuous colorimetric assay using N, N-dimethyl-1,4-phenylenediamine (DMPD) as an electron donor [27, 28]. The assay was performed in a microplate reader (100μl reaction vol.) at pH 5.2, 25 °C in 0.125 M sodium acetate containing 10 mM fumarate, 0.5μM CuSO_4_, 0.1mg/ml catalase (6500 units), 20mM tyramine and 10mM DMPD. A typical reaction used 4μl of serum, which was added to the above reaction mixture without DMPD and was then incubated for 15 min. DMPD was added to initiate the reaction. The absorbance at 515nm was monitored continuously using a UV-VIS spectrometer. A blank control without enzyme was used as background and the slope was subtracted from the measured slopes for enzyme reactions.

### Statistical analysis

Statistical analysis was completed using GraphPad Prism Version 5 (GraphPad Inc., San Diego CA). The chi-square test was used for allele and genotype comparisons of *DβH* polymorphisms between the cases and controls. Differences in DβH levels were analyzed via an unpaired t-test. The effects of different genotypes and alleles on IBD were estimated through odds ratio (OR) and 95% confidence interval (CI). *p*<0.05 was considered as a statistically significant difference.

Sample size was calculated using PASS 16 (NCSS, Inc) software. Given that the anticipated proportion of DβH Arg549Cys in the case group is 0.13, the anticipated proportion in the control group is 0.02, and the difference in group proportions is 0.11. Sample sizes of 44 (88 alleles) for the case group and 66 (132 alleles) for the control group (group ratio as 1:1.5) are needed to detect a two-sided 95% confidence interval for the difference in population proportions with a width of 0.15 [41, 42].
